# Supplementary material for: Immediate nuclear accumulation of BMAL1 to regulate cellular circadian clock synchronization
Source: Commun Biol. 2025 Dec 17;9:104. doi: 10.1038/s42003-025-09373-1 (PMC12830586; doi:10.1038/s42003-025-09373-1)
Supplement: Supplementary file 2 — Supplementary Information [file 42003_2025_9373_MOESM2_ESM.pdf]

Supplementary Information

Immediate nuclear accumulation of BMAL1 to regulate cellular circadian clock synchronization

Teruya Tamaru[1*#], Genki Kawamura[2#], Hikari Yoshitane[3,4], Satoshi Koinuma[5], Yoshitaka Fukada[3,4], Atsuhiko Naito[1], Takeaki Ozawa[2*], Ken Takamatsu[1]

[1] Department of Physiology & Advanced Research Center for Medical Science, Toho University School of Medicine, 5-21-16 Ohmori-nishi, Ohta-ku, Tokyo 143-8540, Japan

[2] Department of Chemistry, School of Science, The University of Tokyo, 7-3-1 Hongo, Bunkyo-ku, Tokyo 113-0033, Japan

[3] Circadian Clock Project, Tokyo Metropolitan Institute of Medical Science, 2-1-6 Kamikitazawa, Setagaya-ku, Tokyo 156-8506, Japan

[4] Department of Biological Sciences, School of Science, The University of Tokyo, 7-3-1 Hongo, Bunkyo- ku, Tokyo 113-0033, Japan

[5] Department of Anatomy and Neurobiology, Kindai University Faculty of Medicine, 1-14-1 Miharadai, Minami-ku, Sakai-City, Osaka 590-0197, Japan

[#] These authors contributed equally

[*] Correspondence should be addressed to:

Teruya Tamaru: tetamaru@med.toho-u.ac.jp, Tel: +813 3762 4151

Takeaki Ozawa: ozawa@chem.s.u-tokyo.ac.jp

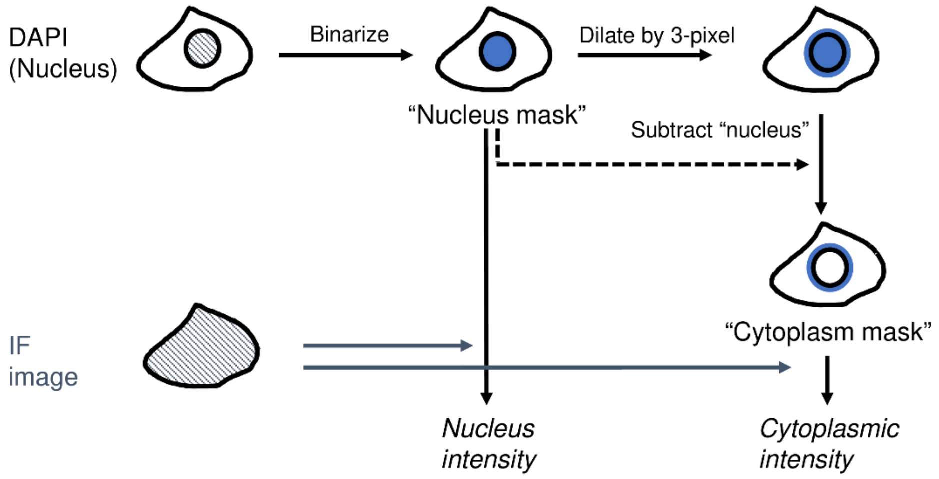

Supplementary Figure 1. Pipeline for evaluating nucleus-to-cytoplasmic fluorescence intensity ratio in immunostaining images.

First, a binarized DAPI or Hoechast33342 image is used to generate a mask image representing the nucleus. The nucleus mask image was dilated by three pixels, and the original nucleus mask image was subtracted from the dilated mask to obtain a “doughnut-shaped” mask, which represents the cytoplasm. These mask images were used to extract the nucleus and cytoplasmic region of the immunostained image. The obtained fluorescence intensities were used to calculate the nucleus-to-cytoplasmic ratio of the cell.

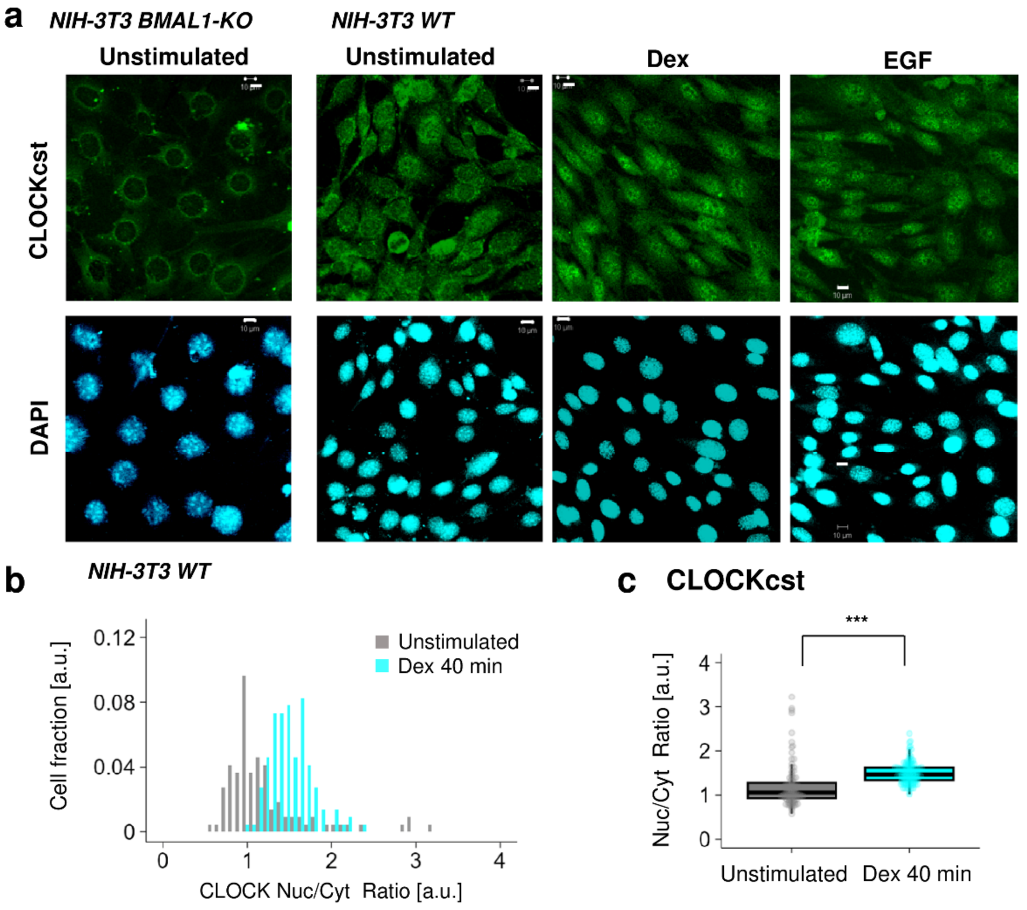

Supplementary Figure 2. Confirmation of immediate nucleocytoplasmic CLOCK changes following clock-synchronization, using ICC with a different antibody.

a Immunofluorescence images of CLOCK protein using rabbit polyclonal antibody. NIH-3T3 (wild-type, WT) and BMAL1-deficient NIH-3T3 (BMAL1-KO) cells were treated with 100 nM dexamethasone (Dex) or 200 ng mL^{-1} EGF 40 min for clock synchronization. or left unstimulated (negative control). Cells were fixed and stained with an anti-CLOCKcst antibody (rabbit polyclonal CST; red) and DAPI (nuclear staining; cyan). Scale bar: 10 µm. **b, c** Quantification of nuclear and cytoplasmic CLOCK levels using a custom Fiji-based analysis procedure as described in the Material and Methods section. Histograms and box-and-whisker plots showed the increase in the nucleocytoplasmic ratio of CLOCK during the initial phase of clock synchronization by Dex. Control: n = 7 independent observation with 97 cells, Dex: n = 5 independent observation with 119 cells. ***: $p < 0.001$, not significant unless mentioned, two-tailed Welch's t-test.

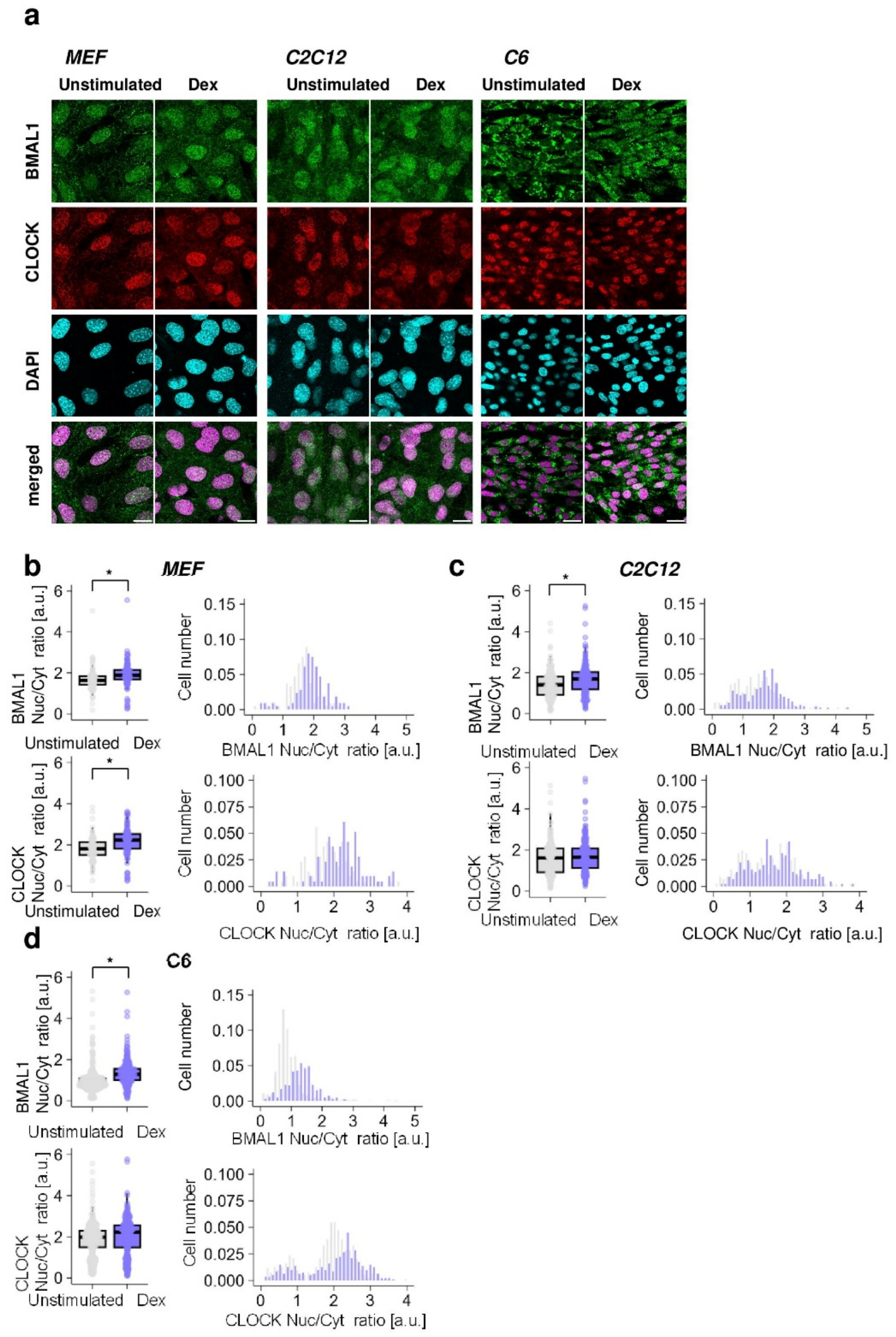

Supplementary Figure 3. Immediate nucleocytoplasmic BMAL1 changes following Dex stimulation for various cell types.

a Representative immunofluorescence image of BMAL1 and CLOCK showing immediate nuclear accumulation of BMAL1. C6, C2C12, and MEF cells were treated with 100 nM dexamethasone (Dex) for 30 min for clock synchronization or left untreated (negative control). Cells were fixed and stained with anti-BMAL1 (rabbit polyclonal Nt; green) and anti-CLOCK (mouse monoclonal CLSP3; red) antibodies, followed by DAPI (nuclear staining; cyan) and

visualized by confocal imaging. The “merged” panel shows merged images of BMAL1 (green) and DAPI (magenta). Scale bars: 10 μm. **b-d** Quantification of nuclear and cytoplasmic BMAL1 and CLOCK levels using a custom Fiji-based analysis procedure as described in Supplementary Figure 1. Quantified results are represented by a box-and-whisker plot and a histogram. MEF, Dex: n = 5 independent observation with 229 cells; unstimulated: n = 4 independent observation with 201 cells, C2C12, Dex: n = 6 independent observation with 437 cells; unstimulated: n = 8 independent observation with 484 cells, C6, Dex: n = 4 independent observation with 672 cells; unstimulated: n = 5 independent observation with 969 cells. *: $p < 0.05$, not significant unless mentioned, two-tailed Welch’s t-test.

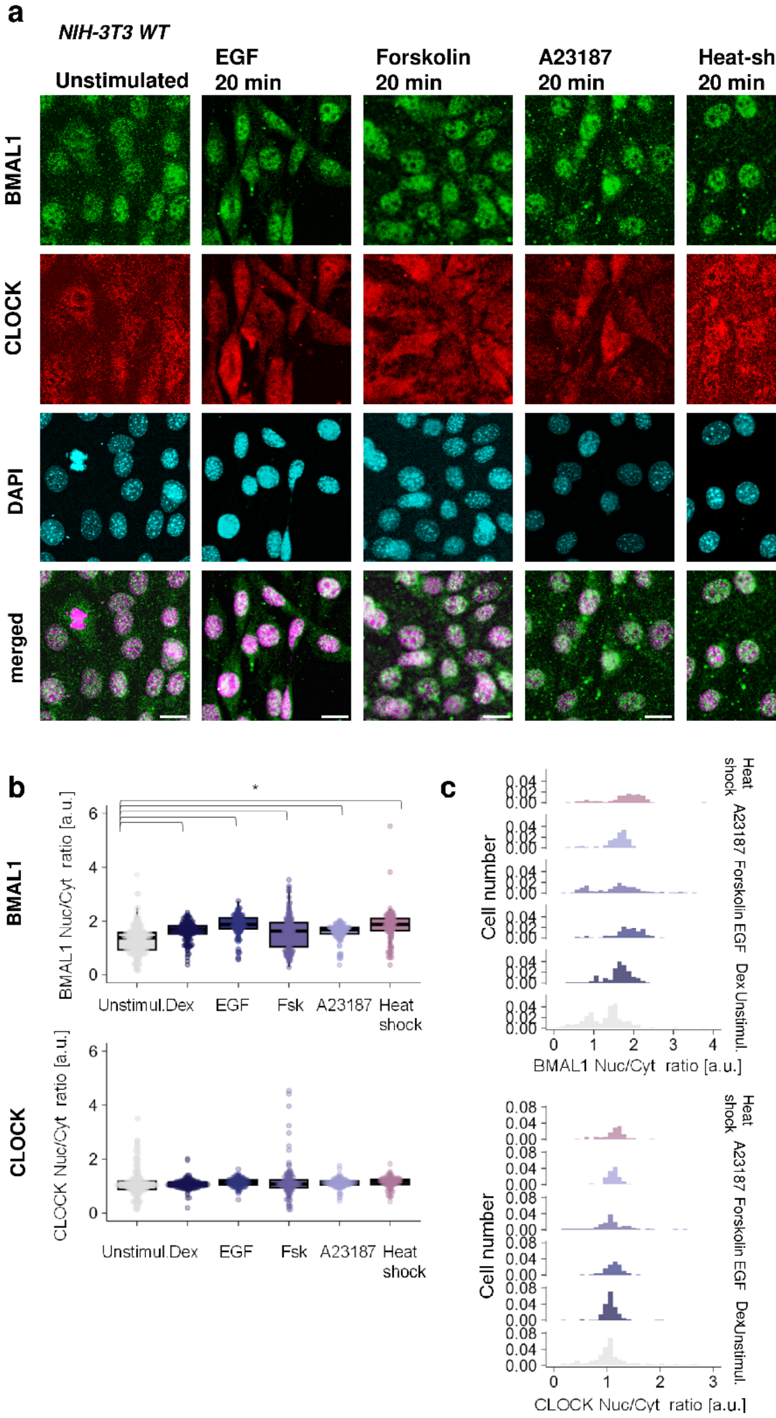

Supplementary Figure 4. Immediate nuclear BMAL1 accumulation following various clock-synchronizing treatments.

a Representative immunofluorescence images of BMAL1 and CLOCK showing immediate nuclear accumulation of BMAL1 followed by CLOCK in NIH-3T3 cells treated with 100 ng mL^{-1} EGF, 10 µM Forskolin, 25 µM A23187 (Ca^{2+} ionophore), or subjected to heat-shock (43°C) for 20 min for clock synchronization. Control cells were left unstimulated. Cells were fixed and stained with anti-BMAL1 and anti-CLOCK antibodies, followed by nuclear staining

with DAPI, and visualized by confocal imaging. Representative images are shown. The “merged” panel shows merged images of BMAL1 (green) and DAPI (magenta). Scale bars: 10 µm. **b, c** Quantification of nuclear and cytoplasmic BMAL1 and CLOCK levels using a custom Fiji-based analysis procedure as described in Supplementary Figure 1. Quantified results are represented by a box-and-whisker plot (b) and histogram (c). Unstimul. Denotes unstimulated samples. Unstimul.: n = 9 independent observations with 568 cells, Dex: n = 5 independent observations with 367 cells, EGF: n = 4 independent observations with 245 cells, Fsk: n = 4 independent observations with 298 cells, A23187: n = 4 independent observations with 259 cells, Heat shock: n = 4 independent observations with 236 cells. *: $p < 0.05$, not significant unless mentioned, Dunnett’s test with p-values adjusted with the Bonferroni method for multiple comparisons.

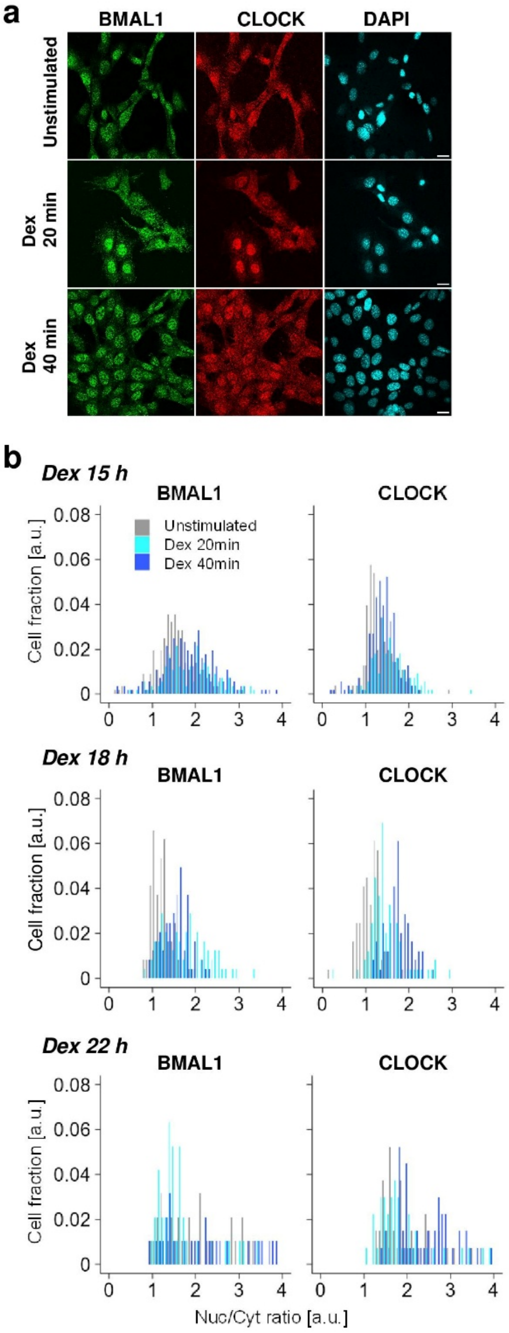

Supplementary Figure 5. Circadian-time-dependent nuclear accumulation of BMAL1.
a Representative immunofluorescence images of BMAL1 and CLOCK protein localization. NIH-3T3 cells were initially treated with Dex for clock synchronization. At circadian time (CT) 15 h, cells were either re-treated with Dex for 20 or 40 min to reset the clock or left unstimulated. Cells were then fixed and stained with anti-BMAL1 and anti-CLOCK antibodies, followed by nuclear staining with DAPI or Hoechst33342, and visualized by confocal imaging. Scale bar: 10 µm. Corresponds to Fig. 2a. **b** Histogram representation of the nuclear-to-cytoplasmic ratio of BMAL1. Data with a Nuc/Cyt ratio between 0 and 4 are shown to

emphasize the distribution changes upon Dex treatment. Corresponds to the data on (a) and Fig. 2a.

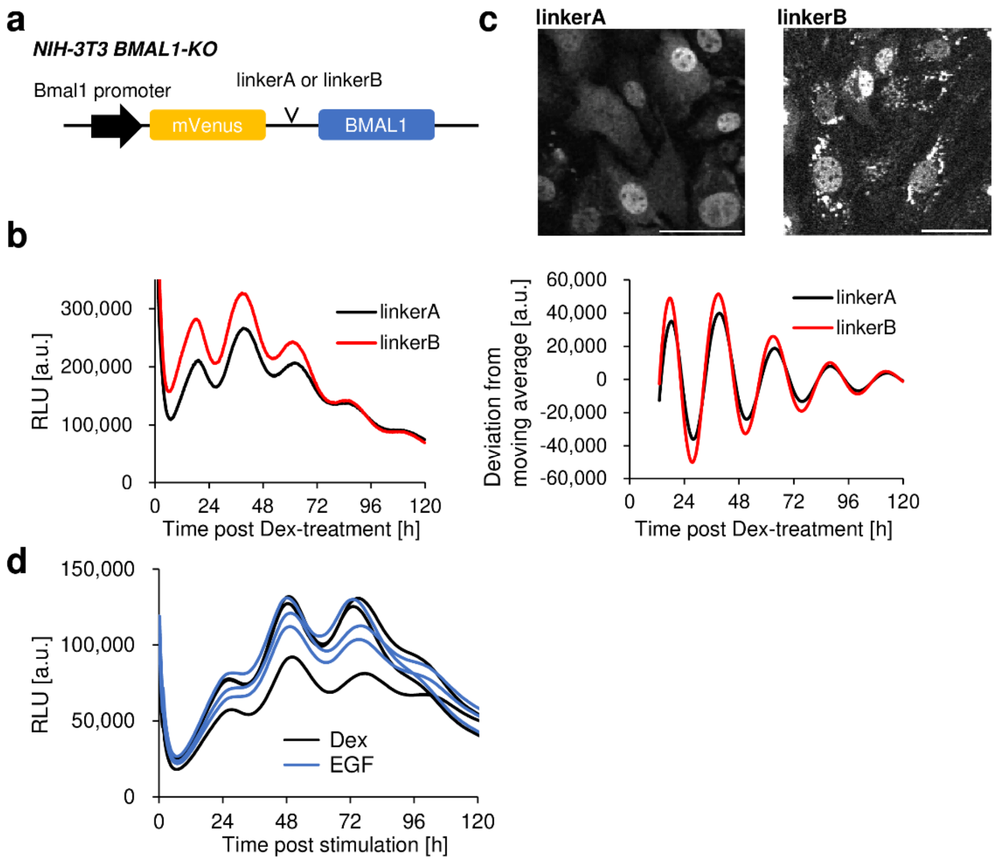

Supplementary Figure 6. Comparison of different peptide linkers for mVenus-BMAL1 reporter.

a A schematic of the BMAL1 reporter. BMAL1 fused with fluorescent protein mVenus (mVenus-BMAL1) is expressed under the control of the *Bmal1* promoter. Two different flexible linker sequences (linkerA and linkerB) were used to connect BMAL1 and mVenus. **b** NIH-3T3 BMAL1-KO cells harboring mVenus-linkerA-BMAL1 and mVenus-linkerB-BMAL1 were stimulated by Dex (100 nM), and the temporal variation of Bmal1-Luc expression was monitored by bioluminescence. Representative traces from three experiments are shown. **c** Representative images of NIH-3T3 BMAL1-KO cells harboring mVenus-linkerA-BMAL1 and mVenus-linkerB-BMAL1 under unstimulated conditions. Scale bar: 50 µm. **d** NIH-3T3 BMAL1-KO cells harboring mVenus-linkerA-BMAL1 and Bmal1-Luc reporter were stimulated with Dex (100 nM) or EGF (100 ng mL^{-1}), and the temporal variation of Bmal1-Luc expression was monitored by a bioluminescence assay. Representative raw traces from three independent experiments are shown. Corresponds to Fig. 3a.

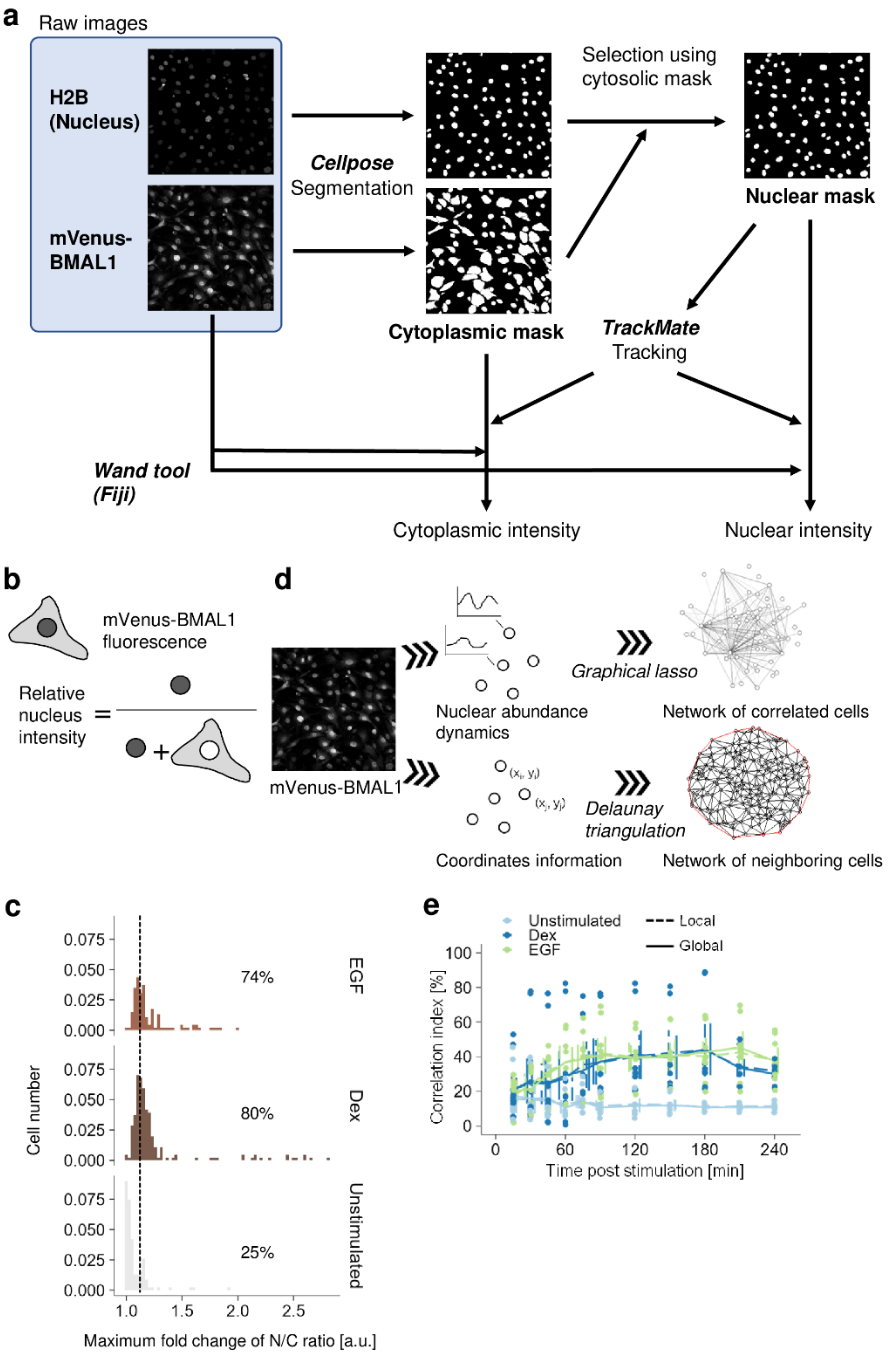

Supplementary Figure 7. Analytical workflow for quantifying nucleus and cytoplasmic fluorescence intensities of mVenus-BMAL1 reporter.

a First, the nucleus and cytoplasm were segmented by Cellpose [33] using mVenus-BMAL1 and H2B-mKate2 images to create a mask image of the nucleus and cytoplasm. To eliminate cells that have no fluorescence from either of the reporters, the selection of the cells was performed using created mask images. A nucleus mask image was used for time-lapse cell tracking using TrackMate [34] and coordinate information was used to measure fluorescence intensities within the mask region in each cell that corresponds to the nucleus or the cytoplasm using the Wand

tool in Fiji software [35]. **b** The degree of nuclear localization of BMAL1 was estimated by calculating the ratio of nuclear fluorescence intensity to the sum of nuclear and cytoplasmic fluorescence intensities. **c** Histogram representation of the maximum fold change of relative nuclear intensity compared to that at t = 0. The dotted line indicates a threshold for positive cells, which was defined as the 75th percentile of the maximum fold change of relative nuclear intensity of the Unstimulated sample. **d** A schematic analytical workflow for calculating the "local correlation". Using the tracked temporal intensity changes of the BMAL1 reporter, the correlation among cellular BMAL1 reporter profiles is calculated with the graphical lasso algorithm [36]. The coordinate information is used to define neighboring cells by the Delaunay triangulation algorithm [37]. **e** Time evolution of correlation indices for Unstimulated, Dex, and EGF stimulated samples. The dotted line indicates a local correlation index and the solid line indicates a global correlation index. Unstimulated: n = 4 independent observations, Dex: n = 4 independent observations, and EGF: n = 4 independent observations. Error bars: standard error.

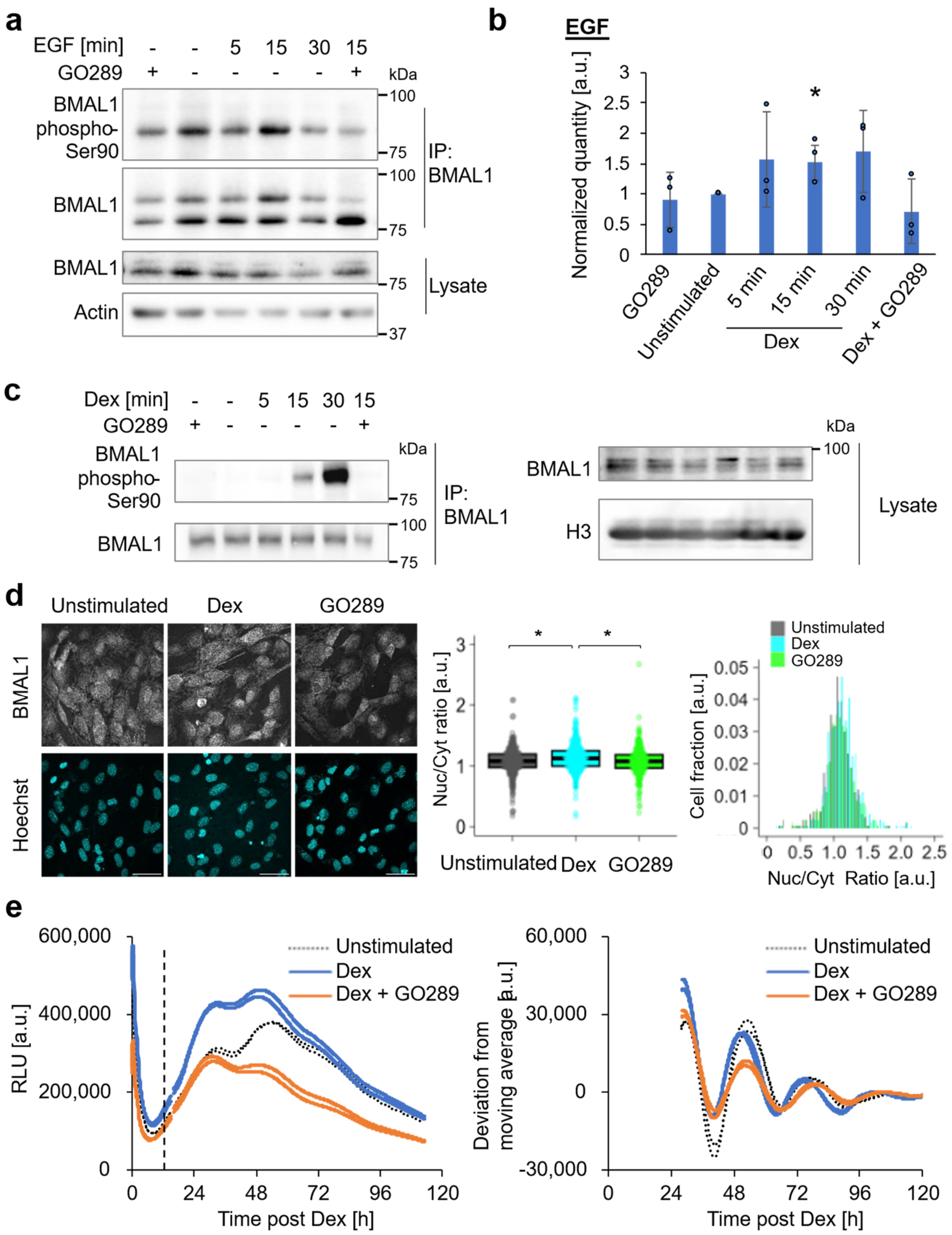

f

	Period [h]	Amplitude [a.u.]	Phase shift from "Unstimulated" [h]	
**Unstimulated**	24.75 ± 0.20	10,084 ± 1,758		n = 4
**Dex**	24.33 ± 1.44	13,233 ± 1,503	-2.14 ± 1.00	n = 5
**Dex + GO289**	24.33 ± 0.60	10,359 ± 1,927	-1.44 ± 0.89	n = 5

Supplementary Figure 8. CK2 inhibition suppresses EGF- and Dex-induced BMAL1 ISR
a Representative immunoblot images of BMAL1-Ser90 phosphorylation bands. Cells were stimulated with EGF in the presence or absence of CK2 inhibitor, GO289, and sampled for immunoprecipitation using an anti-BMAL1 antibody at the indicated time points. See Supplementary Figure 17a for full-size blot images. **b** Quantification of BMAL1-Ser90 phosphorylation levels. Phosphorylation levels were normalized to the total BMAL1 protein

levels in each immunoprecipitated sample and further normalized to the unstimulated control. Actin was used as a loading control. Data represents the mean of three independent experiments. Error bar: SD, *: $p < 0.05$, two-tailed t-test. **c** Representative immunoblots for nuclear BMAL1-Ser90 phosphorylation band. Cells were stimulated with Dex in the presence or absence of CK2 inhibitor, GO289, and sampled for nuclear fractionation, followed by immunoprecipitation using an anti-BMAL1 antibody at the indicated time points. See Supplementary Figure 17b for full-size blot images. **d** Representative immunofluorescence images of BMAL1 localization after Dex stimulation. NIH-3T3 cells were treated with Dex for clock synchronization. At 15 h post Dex stimulation, cells were re-treated with Dex in the presence or absence of CK2 inhibitor, GO289, for 20 min. Cells were then fixed and stained with anti-BMAL1 antibody. Nuclear and cytoplasmic BMAL1:CLOCK levels were quantified using Fiji-based analysis. Box plots and histograms represent the distribution of the nucleus-to-cytoplasmic ratio. In the histogram, data with a Nuc/Cyt ratio of 0 to 2 are shown to emphasize the distribution changes upon Dex treatment. All the data are included in the box plot, with each dot representing each cell analyzed. Scale bar: 50 μm. *: $p < 0.05$, Tukey HSD test. **e** NIH-3T3 BMAL1-KO cells harboring mVenus-linkerA-BMAL1 and Bmal1-Luc reporters were pre-synchronized with Dex. At 15h post-Dex stimulation, cells were restimulated with Dex in the presence (“Dex + GO289”) or absence (“Dex”) of CK2 inhibitor, GO289, or left unstimulated. Cells were transiently stimulated for 60 min and returned to the original medium after stimulation. The temporal luminescence profile of Bmal1-Luc was monitored. Representative Bmal1-Luc profiles, and normalized Bmal1-Luc profiles calculated by subtracting the moving average values, are shown. The dotted line indicates the time point when cells were re-stimulated. **e** Period and amplitude of the Bmal1-Luc luminescence profile following the second Dex stimulation. There are at least 4 replicates for each condition. +/-: SD.

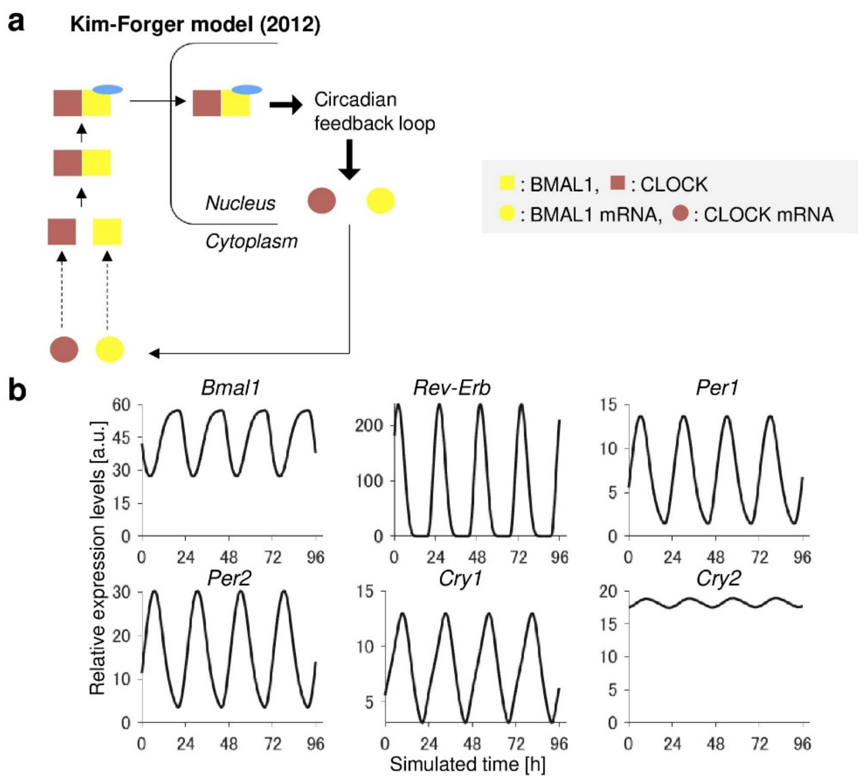

Supplementary Figure 9. Simulation of circadian oscillation with BMAL1 phosphorylation rate modulated.

a Schematic diagram showing the original Kim-Forger model [21] used in this study. The model incorporates modification to include the rate constant change of BMAL1 phosphorylation during synchronization. Only the molecular species that are directly related to BMAL1 nuclear translocation are shown. **b** Simulated circadian expression profiles of *Bmal1, Rev-Erb, Per1, Per2, Cry1,* and *Cry2* generated using the modified model.

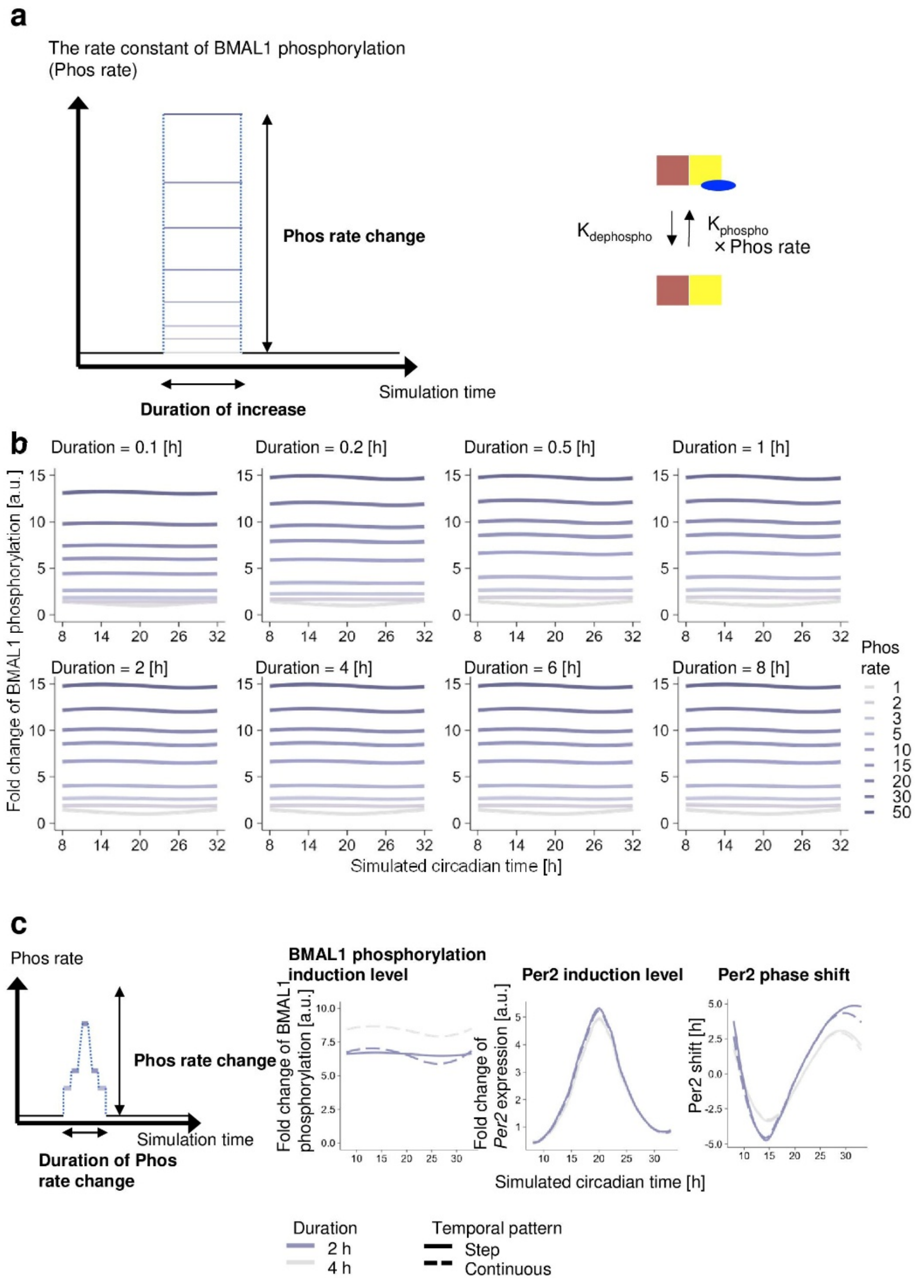

Supplementary Figure 10. Simulation of BMAL1 phosphorylation level changes when BMAL1 phosphorylation rate was modulated.

a Schematics of simulated variables. The rate constant of BMAL1 phosphorylation was increased by “Phos rate change” for a certain duration of Phos rate increase (“Duration of increase”). **b** Simulation result of fold change of BMAL1 phosphorylation. The rate constant of BMAL1 phosphorylation was increased by “Phos rate” for a duration indicated at each panel. The maximum fold change after increasing the rate constant of BMAL1 phosphorylation at each circadian time is shown. **c** Examination of the temporal pattern of phosphorylation rate increase

to BMAL1 phosphorylation level induction, *Per2* expression level induction, and *Per2* phase shift compared to simulation without phosphorylation level increase.

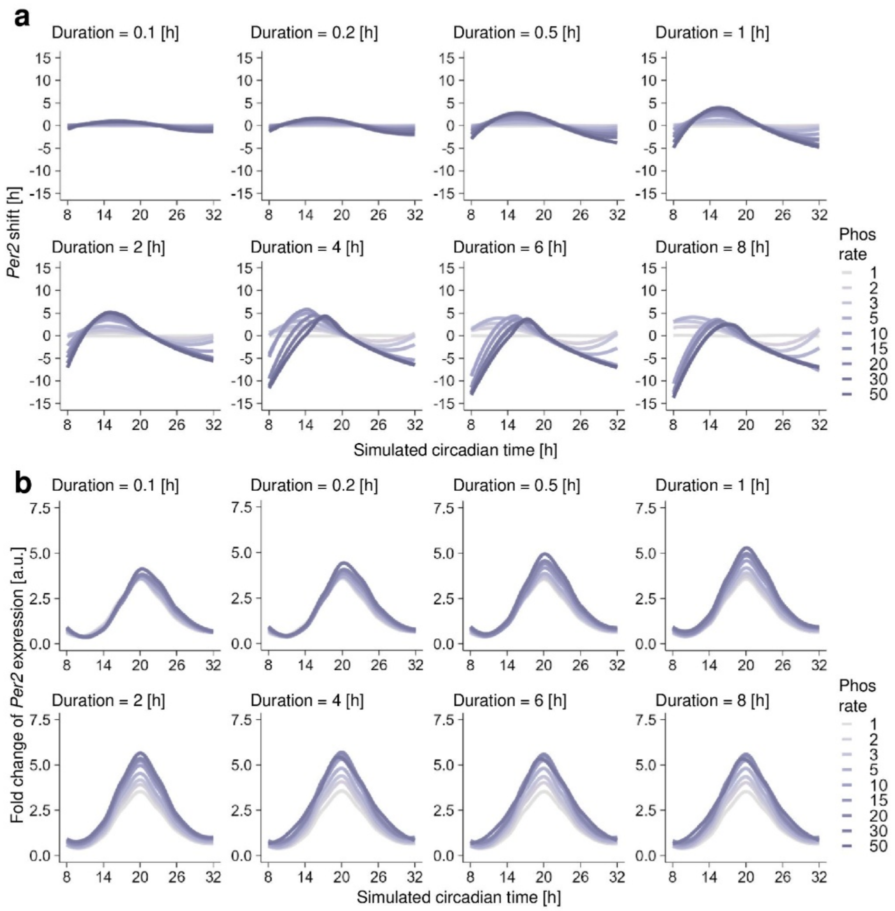

Supplementary Figure 11. Simulation of *Per2* induction and phase shift when BMAL1 phosphorylation rate was modulated.

a, b Simulation result of the effect of a transient increase in the rate constant of BMAL1 phosphorylation on *Per2* expression. The rate constant of BMAL1 phosphorylation was increased by “Phos rate” for a duration indicated at each panel. *Per2* phase shift compared to simulation without phosphorylation level increase (a), and the maximum fold change of *Per2* level (b) after increasing the rate constant of BMAL1 phosphorylation at each circadian time is shown.

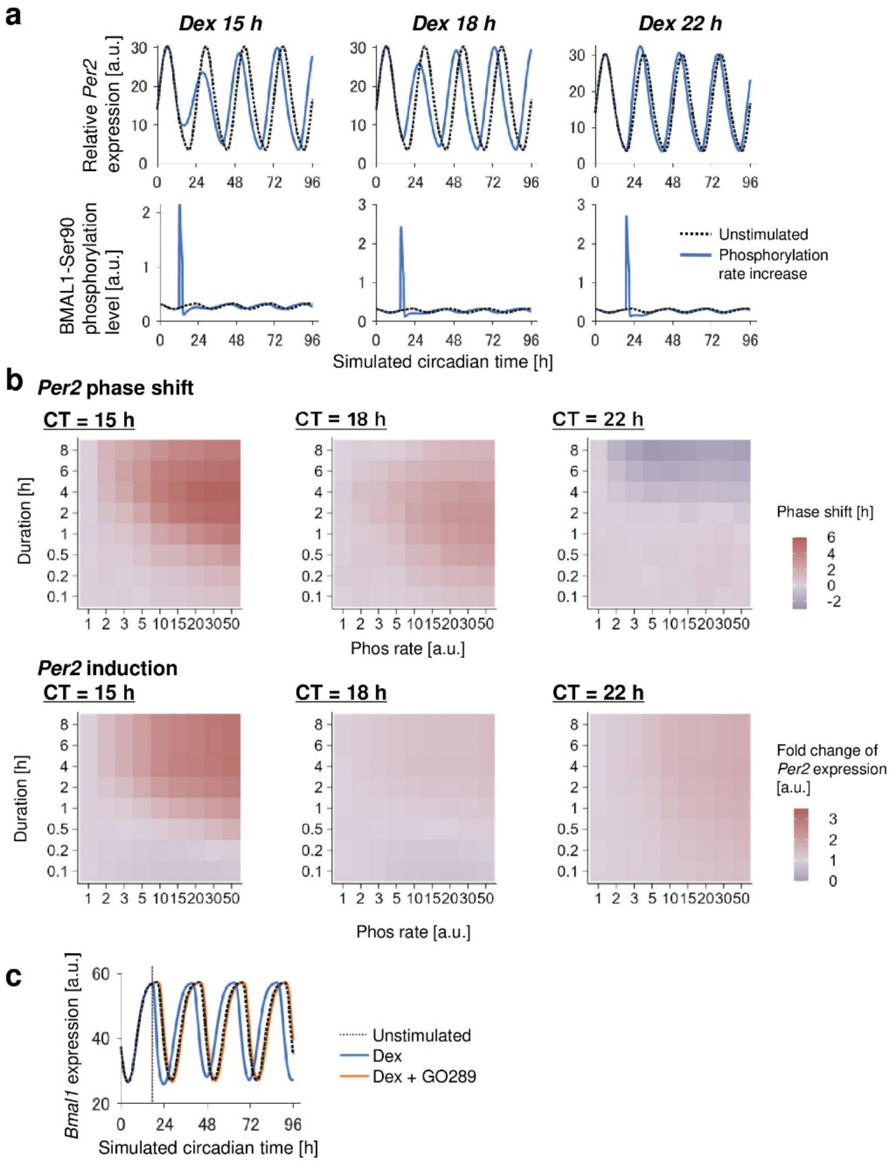

Supplementary Figure 12. Simulation of *Per2* induction and phase shift when BMAL1 phosphorylation rate was modulated.

a Simulated phase shift of *Per2* expression and BMAL1 phosphorylation levels induced by a transient increase in the BMAL1 phosphorylation rate. Simulations were performed with the phosphorylation rate increase applied at 15, 18, and 22 h post- Dex stimulation. **b** Heatmap representation of *Per2* phase shift and maximum fold change of *Per2* expression change after an increase in the rate constant of BMAL1 phosphorylation with different Phos rate change and duration. Simulations were performed with the phosphorylation rate increase applied at 15, 18, and 22 h post-Dex stimulation**. c** Simulation of Bmal1-Luc in response to CK2 inhibition by GO289. Dex stimulation was modeled as an increase in BMAL1 phosphorylation rate for a duration of 2 hours. GO289 treatment was modeled as a decrease in the BMAL1 phosphorylation rate for 2 hours. The dotted line indicates the time point when Dex ("Dex") or a

combination of Dex and GO289 (“Dex + GO289”) were applied. “Unstimul” denotes a simulation without Dex or GO289 treatment.

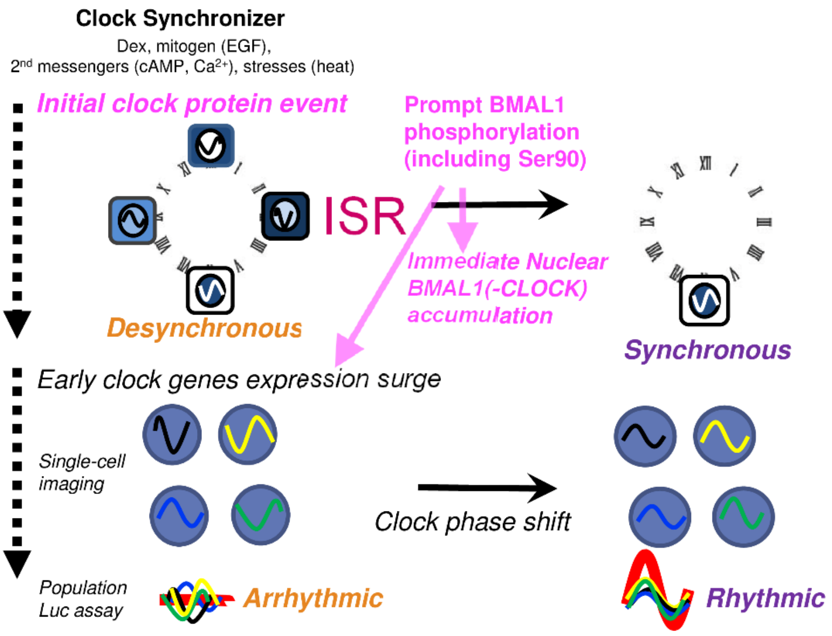

Supplementary Figure 13. A proposed model for BMAL1-ISR as one of the switching signals that links internal clock oscillation and synchronization.

A schematic model illustrating how BMAL1-ISR may function as a key signaling pathway linking internal circadian oscillations with external synchronizing cues. In the hypothesis, the distribution of nucleocytoplasmic BMAL1 localization reflects circadian time.

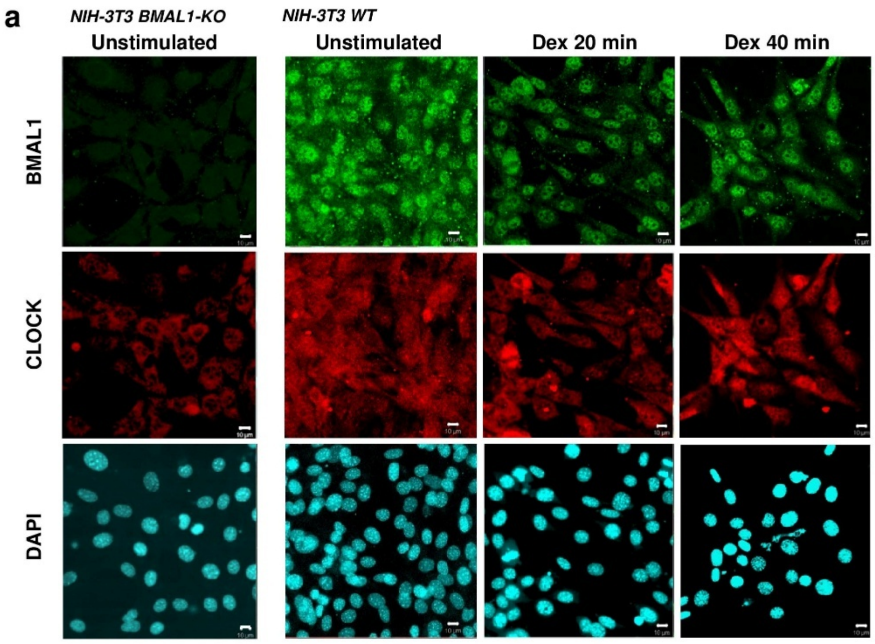

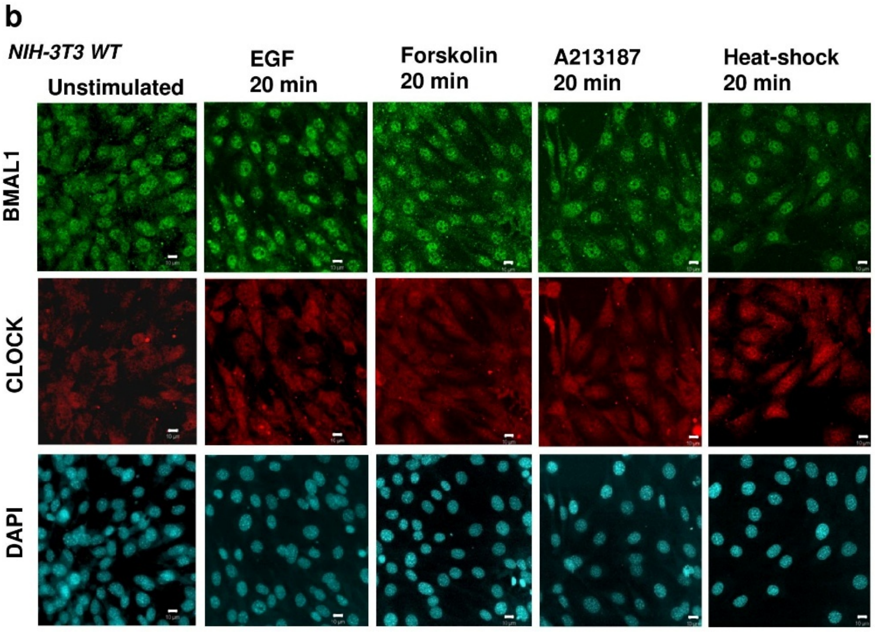

Supplementary Figure 14. Uncropped images for Fig. 1a and Supplementary Figure 4

Uncropped images representing cells with various spatial distribution of BMAL1 and CLOCK corresponding to Fig. 1a (a) and Supplementary Figure 4a (b).

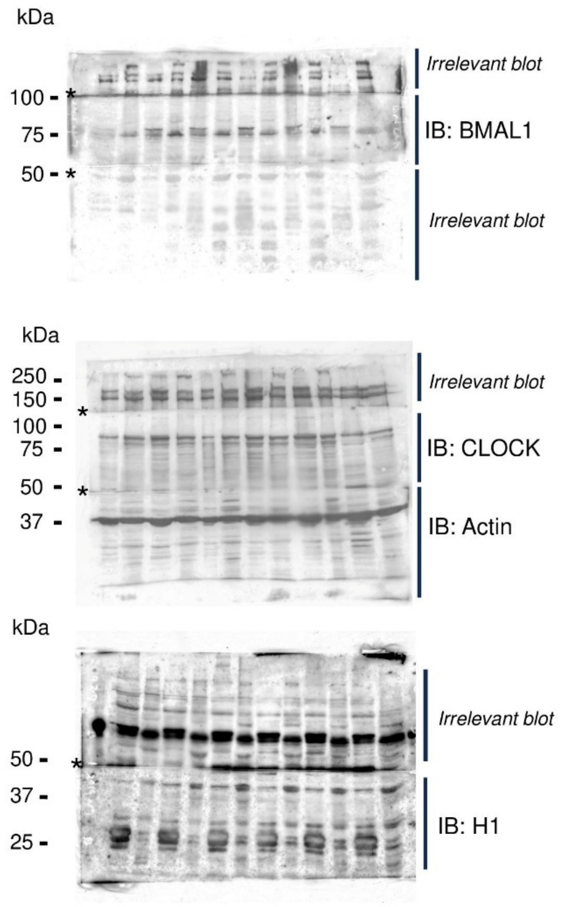

Supplementary Figure 15. Full-sized blot for Fig. 2d

a-c Full-sized immunoblots corresponding to Fig. 2d. The membranes were cut at 100 kDa and 50 kDa positions (indicated with *) corresponding to the marker to enable detection of multiple targets with different expected molecular weights, including BMAL1 (a), CLOCK, and Actin (b). The membrane was cut at the 50 kDa position (indicated with *) corresponding to the marker to detect H1 (c).

a

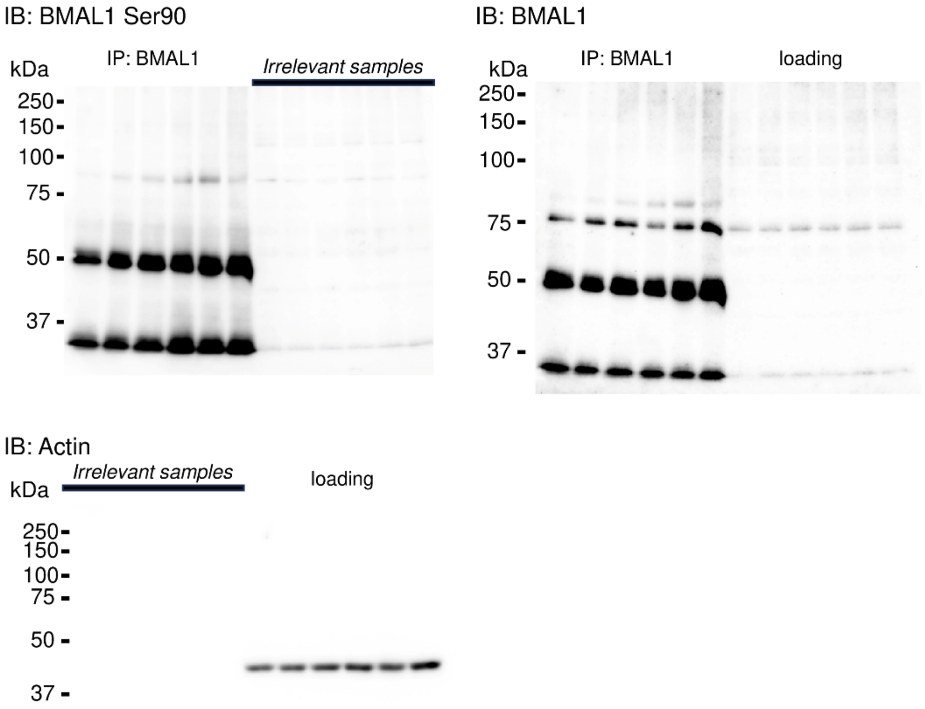

b

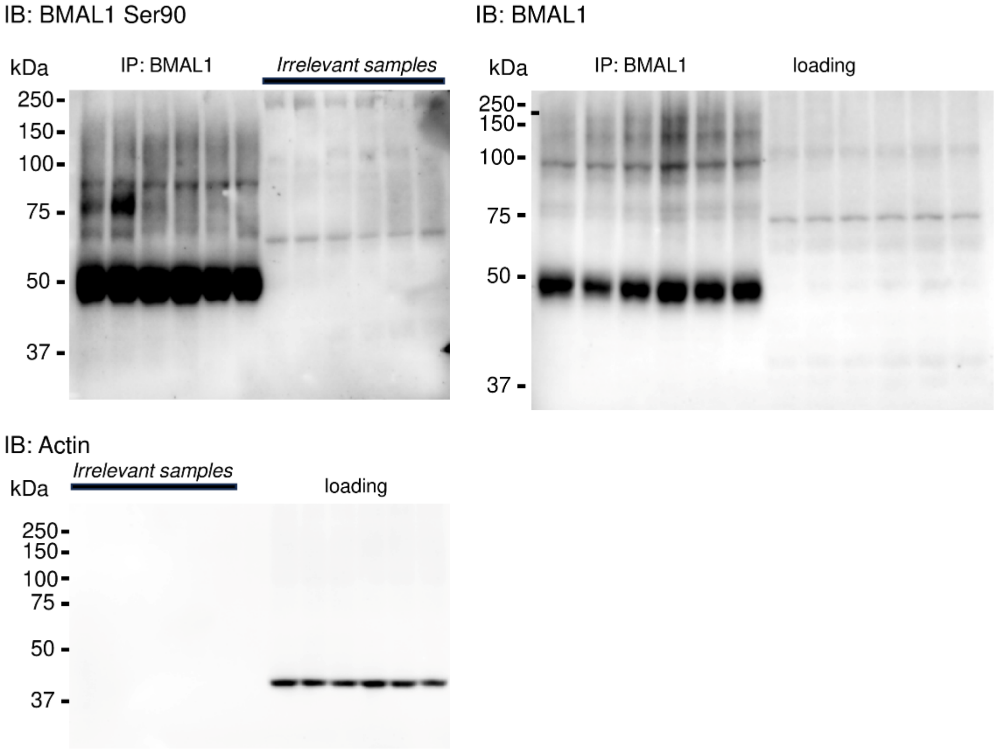

Supplementary Figure 16. Full-sized blot for Fig. 4a

a, b Full-sized immunoblots corresponding to Fig. 4a. Immunoblots for Dex 15 h (**a**) and Dex 22 h (**b**) samples are shown. 'IP: BMAL1' denotes the bands for immunoprecipitated samples

with anti-BMAL1 antibody. ‘loading’ denotes that the bands are used for the loading control. The sign “irrelevant samples” on the top of the bands indicates that these bands were not used in this study.

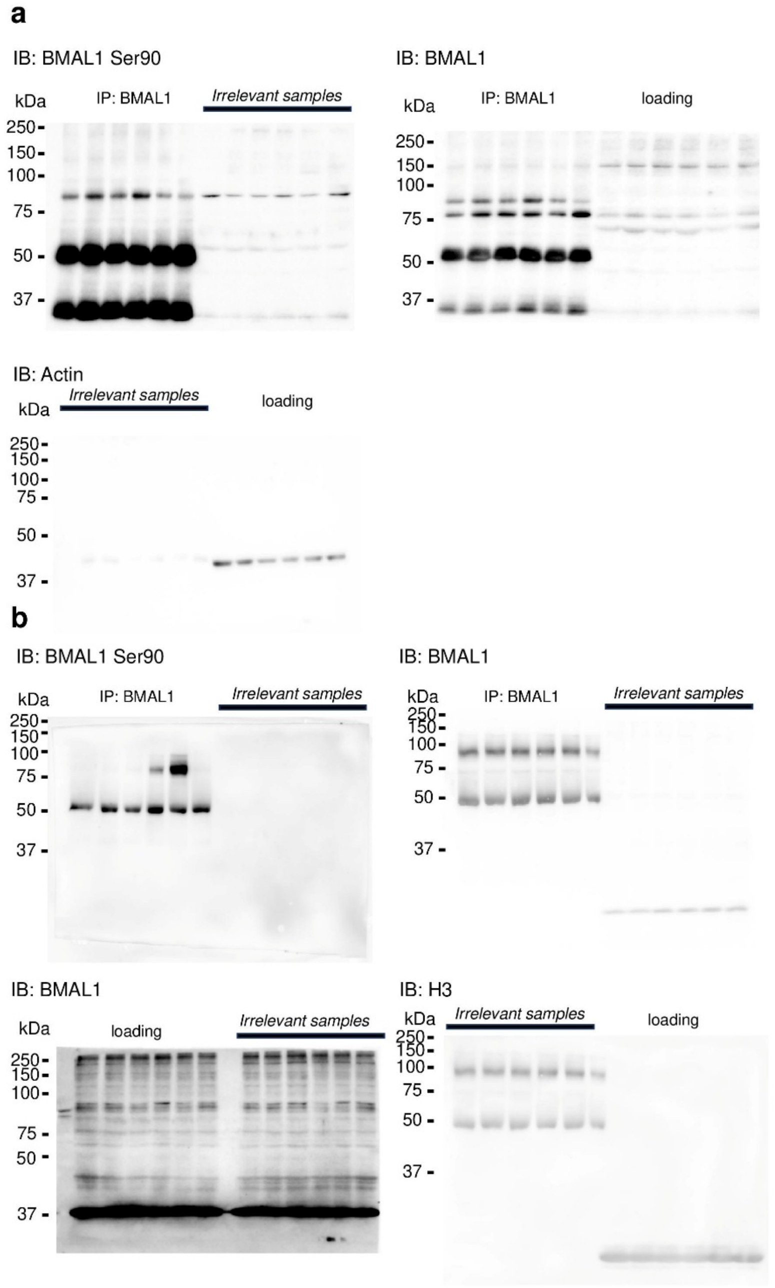

Supplementary Figure 17. Full-sized blots for Supplementary Figure 8

a, b Full-sized immunoblots corresponding to Supplementary Figure 8a and c. 'IP: BMAL1' denotes the bands for immunoprecipitated samples with anti-BMAL1 antibody. 'loading' denotes that the bands are used for the loading control. The sign "irrelevant samples" on the top of the bands indicates that these bands were not used in this study. We note that H3 (lower right, (b)) was detected after reprobing of BMAL1 (upper right, (b)), thus the same membrane was used for the detection of these two targets.

Supplementary Table 1. List of exact p values

Fig. 1c BMAL1 and CLOCK nuclear accumulation upon Dex stimulation.

Statistical test: Tukey's honestly significant difference test

Exact p values:

	**BMAL1**	**CLOCK**
Unstimulated v.s. Dex 20 min	3.890×10^{-8} *	0.2406
Unstimulated v.s. Dex 40 min	3.395×10^{-7} *	1.210×10^{-6} *
Dex 20 min v.s. Dex 40 min	0.2323	3.142×10^{-5} *

Fig. 2b Circadian-time dependent BMAL1 and CLOCK nuclear abundance.

Statistical test: Tukey's honestly significant difference test

Exact p values:

	**BMAL1**	**CLOCK**
15 h v.s. 18 h	2.2×10^{-15} *	1.856×10^{-7} *
15 h v.s. 22 h	1.434×10^{-7} *	1.434×10^{-7} *
18 h v.s. 22 h	4.570×10^{-5} *	1.505×10^{-12} *

Fig. 2c Circadian-time dependent response to Dex stimulation.

Statistical test: Tukey's honestly significant difference test

Exact p values:

BMAL1

	**15 h**	**18 h**	**22 h**
Unstimulated v.s. Dex 20 min	1.393×10^{-8} *	1.321×10^{-13} *	0.9982
Unstimulated v.s. Dex 40 min	3.728×10^{-7} *	2.071×10^{-4} *	0.4058
Dex 20 min v.s. Dex 40 min	0.3893	1.121×10^{-4} *	0.3431

CLOCK

	**15 h**	**18 h**	**22 h**
Unstimulated v.s. Dex 20 min	5.248×10^{-7} *	4.990×10^{-11} *	0.3675
Unstimulated v.s. Dex 40 min	0.4889	9.182×10^{-14} *	5.694×10^{-2}
Dex 20 min v.s. Dex 40 min	3.806×10^{-5} *	3.701×10^{-6} *	0.5851

Fig. 2e Dex-induced accumulation of BMAL1 in the nuclear fraction.

Statistical test: Welch Two Sample t-test

Exact p values:

Dex 15 h	1.898×10^{-2} *
Dex 18 h	0.2435
Dex 22 h	0.1687

Fig. 3c Time evolution of nuclear/cytoplasmic ratio.

Statistical test: Tukey's honestly significant difference test

Exact p values:

Time point	Unstimulated	Dex	EGF
0.3 h	0.982	1.264×10^{-2} *	1.479×10^{-2} *
0.6 h	0.825	3.932×10^{-5} *	1.018×10^{-4} *
0.9 h	0.796	3.164×10^{-5} *	5.401×10^{-5} *
1.2 h	0.851	3.761×10^{-4} *	1.424×10^{-5} *
1.5 h	0.705	4.884×10^{-3} *	8.807×10^{-6} *

Fig. 3f Correlation index

Statistical test: Tukey's honestly significant difference test

Exact p values:

Local correlation index

Unstimulated v.s. Dex	8.222×10^{-3} *
Unstimulated v.s. EGF	3.974×10^{-3} *
Dex v.s. EGF	0.8725

Global correlation index

Unstimulated v.s. Dex	5.661×10^{-3} *
Unstimulated v.s. EGF	8.152×10^{-3} *
Dex v.s. EGF	0.9666

Fig. 4b BMAL1 Ser90 phosphorylation level change upon stimulation.

Statistical test: Tukey's honestly significant difference test

Exact p values:

	Dex 15h	Dex 22h
GO289	0.08754	0.3553
5 min post Dex	0.1631	0.2129
15 min post Dex	0.04363 *	0.07865
30 min post Dex	0.002250 *	0.06460
Dex + GO289	0.9063	0.5921

Fig. 4d Circadian oscillation period, amplitude and phase upon Dex stimulation

Statistical test: Tukey's honestly significant difference test

Exact p values:

Condition	Period	Amplitude	Phase-shift
Unstimulated v.s. Dex	0.1561	7.163×10^{-3} *	1.287×10^{-5} *
Unstimulated v.s. Dex + GO289	0.1279	0.7654	0.7656
Dex v.s. Dex + GO289	0.9899	1.382×10^{-2} *	1.398×10^{-6} *

Fig. 5d qPCR measurement of *Per2* expression

Statistical test: Tukey's honestly significant difference test

Exact p values:

**Condition**	**15 h**	**18 h**	**22 h**
Unstimulated v.s. Dex	1.590×10^{-4} *	2.585×10^{-4} *	0.2246
Unstimulated v.s. Dex + GO289	0.8997	0.1377	0.3015
Dex v.s. Dex + GO289	2.600×10^{-4} *	2.880×10^{-5} *	0.9691

Supplementary Figures

Supplementary Figure 2c Analysis of CLOCK nuclear accumulation using a different anti-CLOCK antibody

Statistical test: Welch Two Sample t-test

CLOCKcst Unstimulated v.s. Dex: $p = 4.239 \times10^{-7}$ *

Supplementary Figure 3b, c, d Nuclear-to-cytoplasmic ratio change upon Dex stimulation in various cell types.

Statistical test: Welch Two Sample t-test for each cell type and target protein.

Exact p values:

**Cell type**	**BMAL1**	**CLOCK**
MEF	2.806×10^{-3} *	1.407×10^{-4} *
C2C12	0.02469 *	0.2769
C6	1.702×10^{-12} *	0.1185

Supplementary Figure 4b Nuclear-to-cytoplasmic ratio change upon various synchronizer stimulation.

Statistical test: Dunnett's test with p-values adjusted with the Bonferroni method for multiple comparisons

Exact p values:

	**Target protein**	
**Stimulation**	**BMAL1**	**CLOCK**
Dex	3.706×10^{-9} *	0.09007
EGF	2.200×10^{-16} *	0.3264
Forskolin	3.261×10^{-4} *	0.2658
A23187	2.467×10^{-7} *	0.6706
Heat-shock	1.248×10^{-11} *	0.1860

Supplementary Figure 8b Circadian oscillation period, amplitude and phase upon EGF stimulation

	**Dex 15h**
GO289	0.7387
5 min post Dex	0.2756
15 min post Dex	0.04970 *
30 min post Dex	0.1450
Dex + GO289	0.3981

Supplementary Figure 8d Comparison of nuclear-to-cytoplasmic ratio chang upon GO289 treatment

Statistical test: Tukey's honestly significant difference test

Exact p values:

**Condition**	
Unstimulated v.s. Dex	8.630×10^{-5} *
Unstimulated v.s. Dex + GO289	0.9862
Dex v.s. Dex + GO289	3.147×10^{-3} *
