# Supplementary material for: Immediate nuclear accumulation of BMAL1 to regulate cellular circadian clock synchronization
Source: Commun Biol. 2025 Dec 17;9:104. doi: 10.1038/s42003-025-09373-1 (PMC12830586; doi:10.1038/s42003-025-09373-1)
Supplement: Supplementary file 3 — Description of Additional Supplementary Files [file 42003_2025_9373_MOESM3_ESM.pdf]

Description of Additional Supplementary Files

File name: Supplementary Data 1

Description: The numerical source data for the graphs and charts (.xlsx file).

File Name: Supplementary Movie 1

Description: Live-cell imaging of BMAL1 localization change without stimulation. A representative time-lapse imaging of mVenus-linkerA-BMAL1 cells left unstimulated. Corresponds to “Unstimulated” images in Fig. 3b. Frame rate: 15 fps.

File Name: Supplementary Movie 2:

Description: Live-cell imaging of BMAL1 localization change upon Dex stimulation. A representative time-lapse imaging of mVenus-linkerA-BMAL1 cells stimulated with 100 nM Dex. Corresponds to “Dex” images in Fig. 3b. Frame rate: 15 fps.

File Name: Supplementary Movie 3

Description: A representative time-lapse imaging of mVenus-linkerA-BMAL1 cells stimulated with 100 ng mL^{-1} EGF. Corresponds to “EGF” images in Fig. 3b. Frame rate: 15 fps.
