# Supplementary material for: Immediate nuclear accumulation of BMAL1 to regulate cellular circadian clock synchronization
Source: Commun Biol. 2025 Dec 17;9:104. doi: 10.1038/s42003-025-09373-1 (PMC12830586; doi:10.1038/s42003-025-09373-1)
Supplement: Supplementary file 8 — Reporting Summary [file 42003_2025_9373_MOESM8_ESM.pdf]

nature portfolio

Corresponding author(s): Takeaki Ozawa
Teruya Tamaru

Last updated by author(s): Nov 13, 2025

Reporting Summary

Nature Portfolio wishes to improve the reproducibility of the work that we publish. This form provides structure for consistency and transparency in reporting. For further information on Nature Portfolio policies, see our Editorial Policies and the Editorial Policy Checklist.

Statistics

For all statistical analyses, confirm that the following items are present in the figure legend, table legend, main text, or Methods section.

n/a	Confirmed	
☐	☒	The exact sample size (*n*) for each experimental group/condition, given as a discrete number and unit of measurement
☐	☒	A statement on whether measurements were taken from distinct samples or whether the same sample was measured repeatedly
☐	☒	The statistical test(s) used AND whether they are one- or two-sided<br>*Only common tests should be described solely by name; describe more complex techniques in the Methods section.*
☐	☒	A description of all covariates tested
☒	☐	A description of any assumptions or corrections, such as tests of normality and adjustment for multiple comparisons
☐	☒	A full description of the statistical parameters including central tendency (e.g. means) or other basic estimates (e.g. regression coefficient) AND variation (e.g. standard deviation) or associated estimates of uncertainty (e.g. confidence intervals)
☐	☒	For null hypothesis testing, the test statistic (e.g. *F*, *t*, *r*) with confidence intervals, effect sizes, degrees of freedom and *P* value noted<br>*Give P values as exact values whenever suitable.*
☒	☐	For Bayesian analysis, information on the choice of priors and Markov chain Monte Carlo settings
☒	☐	For hierarchical and complex designs, identification of the appropriate level for tests and full reporting of outcomes
☒	☐	Estimates of effect sizes (e.g. Cohen's *d*, Pearson's *r*), indicating how they were calculated

Our web collection on statistics for biologists contains articles on many of the points above.

Software and code

Policy information about availability of computer code

Data collection	The following softwares were used for data collection.<br>LuminoGraphIII Ver. 1.3.8 for Western blot band detection, Kronos Version 2.10.230 for bioluminescence measurement, cellSens Dimension ver. 3.1 for live-cell fluorescence imaging, Fluorview Ver. 4.2c for imaging of immunostained samples, and Thermal Cycle Dice Real Time System Software Ver. 5.11B for qPCR measurements.
Data analysis	The following softwares were used for data analysis.<br>Microsoft Excel 365 for generating graphs, R 4.4.0 and RStudio 2023.06.1+524 for statistical analysis and generating graphs, ImageJ 1.54p for image quantification, and MATLAB 2023b for simulation analysis and generating graphs.

For manuscripts utilizing custom algorithms or software that are central to the research but not yet described in published literature, software must be made available to editors and reviewers. We strongly encourage code deposition in a community repository (e.g. GitHub). See the Nature Portfolio guidelines for submitting code & software for further information.

Data

Policy information about availability of data

All manuscripts must include a data availability statement. This statement should provide the following information, where applicable:

- Accession codes, unique identifiers, or web links for publicly available datasets
- A description of any restrictions on data availability
- For clinical datasets or third party data, please ensure that the statement adheres to our policy

The numerical source data for all the graphs and charts are available on Supplementary Data 1. The codes for the simulation and analysis are deposited at Zenodo [https://doi.org/10.5281/zenodo.17568955]. Plasmids newly generated in this study, along with their maps and nucleotide sequences, will be available at the Addgene repository (#249336, #249337, #249338).

Research involving human participants, their data, or biological material

Policy information about studies with human participants or human data. See also policy information about sex, gender (identity/presentation), and sexual orientation and race, ethnicity and racism.

Reporting on sex and gender	This study does not include human participants or human data.
Reporting on race, ethnicity, or other socially relevant groupings	This study does not include human participants or human data.
Population characteristics	This study does not include human participants or human data.
Recruitment	This study does not include human participants or human data.
Ethics oversight	This study does not include human participants or human data.

Note that full information on the approval of the study protocol must also be provided in the manuscript.

Field-specific reporting

Please select the one below that is the best fit for your research. If you are not sure, read the appropriate sections before making your selection.

☒ Life sciences ☐ Behavioural & social sciences ☐ Ecological, evolutionary & environmental sciences

For a reference copy of the document with all sections, see nature.com/documents/nr-reporting-summary-flat.pdf

Life sciences study design

All studies must disclose on these points even when the disclosure is negative.

Sample size	No statistical sample size calculations were performed.
Data exclusions	No data were excluded for statistical analysis.
Replication	Experimental findings were reliably reproduced in all cases. Each Experiments was performed by applying the same technical protocol.
Randomization	Randomization was not relevant for data included in the manuscript.
Blinding	The investigators were not blinded to allocation during experiments and outcome assessment.

Reporting for specific materials, systems and methods

We require information from authors about some types of materials, experimental systems and methods used in many studies. Here, indicate whether each material, system or method listed is relevant to your study. If you are not sure if a list item applies to your research, read the appropriate section before selecting a response.

Materials & experimental systems

n/a	Involved in the study	
☐	☒	Antibodies
☐	☒	Eukaryotic cell lines
☒	☐	Palaeontology and archaeology
☒	☐	Animals and other organisms
☒	☐	Clinical data
☒	☐	Dual use research of concern
☒	☐	Plants

Methods

n/a	Involved in the study	
☒	☐	ChIP-seq
☒	☐	Flow cytometry
☒	☐	MRI-based neuroimaging

Antibodies

Antibodies used	Primary antibodies for BMAL1 (custom made), phospho-Ser90 BMAL1 (custom made), CLOCK (#5157, Cell Signaling Technology), CLOCK (#D333-3, MBP), beta-actin (AC-15, Sigma), and histones (#MAB052, Chemicon) were used. Anti-BMAL1 and anti-phospho-Ser90-BMAL1 antibodies were previously made in our laboratory. For Secondary antibodies, HRP-conjugated anti-mouse or anti rabbit IgG secondary antibodies (GE healthcare), AlexaFluor488 conjugated anti-rabbit IgG (1:250, # A-11008, Thermo fisher) , AlexaFluor647 conjugated anti-rabbit IgG (1:1,000, #A-21245, Thermo fisher), and AlexaFlour568 conjugated anti-Mouse IgG (1:1,000, #A-11004, Thermo fisher) were used.
Validation	All antibodies in this study were validated first by the manufacturer, except for the anti-BMAL1 and anti-phospho-Ser90-BMAL1 antibodies which were previously made and validated in our lab (Tamaru et al. 2003 and Tamaru et al. 2009, respectively).

Eukaryotic cell lines

Policy information about cell lines and Sex and Gender in Research

Cell line source(s)	Mouse fibroblast NIH-3T3 cell (RIKEN cell bank, Japan), C6 cell (RIKEN Cell Bank, Japan), mouse fibroblast MEF cell (obtained from Dr. Christopher A. Bradfield), mouse myoblast C2C12 cell (CRL-1772, ATCC), and plat-E cells (Cell Biolabs Inc.).
Authentication	The clonal NIH-3T3 cells were characterized in previous studies (Tamaru et al. (2015) and Abe et al. (2022)). Morphology and behavior were consistent with their identity but genetic validation was not carried out.
Mycoplasma contamination	Cell lines were tested for mycoplasma contamination by PCR and found negative.
Commonly misidentified lines (See ICLAC register)	No commonly used misidentified cell lines were used.

Plants

Seed stocks	This study does not include experiment using plants.
Novel plant genotypes	This study does not include experiment using plants.
Authentication	This study does not include experiment using plants.
